# The impact of the COVID-19 pandemic on diagnosis and treatment of patients with soft tissue and bone sarcomas or aggressive benign musculoskeletal diseases: A single-center retrospective study (SarCorD study)

**DOI:** 10.3389/fonc.2022.1000056

**Published:** 2022-09-20

**Authors:** Concetta Elisa Onesti, Sabrina Vari, Francesca Nardozza, Gabriella Maggi, Denise Minghelli, Barbara Rossi, Francesca Sperati, Elisa Checcucci, Wioletta Faltyn, Maria Cecilia Cercato, Antonella Cosimati, Roberto Biagini, Gennaro Ciliberto, Virginia Ferraresi

**Affiliations:** ^1^ Sarcomas and Rare Tumors Unit, Istituti di Ricovero e Cura a Carattere Scientifico (IRCCS) Regina Elena National Cancer Institute, Rome, Italy; ^2^ Unità Operativa Semplice Dipartimentale (UOSD) Clinical Trial Center, Biostatistics and Bioinformatics, IRCCS Regina Elena National Cancer Institute, Rome, Italy; ^3^ Psychology Unit, IRCCS Regina Elena National Cancer Institute, Rome, Italy; ^4^ Oncological Orthopaedics Unit, IRCCS Regina Elena National Cancer Institute, Rome, Italy; ^5^ UOSD Clinical Trial Center, Biostatistics and Bioinformatics, IRCCS San Gallicano Dermatological Institute, Rome, Italy; ^6^ Epidemiology and Tumor Registry Unit, IRCCS Regina Elena National Cancer Institute, Rome, Italy; ^7^ Department of Clinical and Molecular Medicine, Sapienza University of Rome, Rome, Italy; ^8^ Scientific Direction, IRCCS Regina Elena National Cancer Institute, Rome, Italy

**Keywords:** COVID-19, pandemic, soft tissue sarcoma, bone sarcoma, aggressive benign musculoskeletal diseases, diagnostic delay

## Abstract

**Background:**

The COVID-19 pandemic led to a rapid reorganization of healthcare activities, leading to reduced access to clinics, interruption of screenings, and treatment schedule modifications in several cancer types. Few data are available on sarcomas. We analyzed COVID-19-related diagnostic delay in a sarcoma referral center in Italy.

**Methods:**

We retrospectively enrolled in this study patients with histological diagnosis of soft tissue or bone sarcoma and aggressive benign musculoskeletal diseases obtained during the first year of the pandemic (Covid group) or the year before (Control group) and followed at the Regina Elena National Cancer Institute in Rome. The primary endpoint was the time from the first symptom to histological diagnosis.

**Results:**

We evaluated 372 patients, 185 of whom were eligible for primary endpoint analysis (92 patients in the Control group and 93 patients in the Covid group). The patients were affected by soft tissue sarcoma in most cases (63.0% and 66.7% in Covid and Control groups, respectively). We observed a diagnostic delay in the Covid group with a median time from the first symptom to the definitive histological diagnosis of 103.00 days (95% CI 92.77–113.23) *vs*. 90.00 days (95% CI 69.49–110.51) in the Control group (*p* = 0.024), but not a delay in treatment beginning (151 days, 95% CI 132.9–169.1 *vs*. 144 days, 95% CI 120.3–167.7, respectively, *p* = 0.208). No differences in stage at diagnosis were observed (12% *vs*. 16.5% of patients with metastatic disease at diagnosis in the Covid and Control groups, respectively, *p* = 0.380). Progression-free survival (*p* = 0.897) and overall survival (*p* = 0.725) were comparable in the subgroup of patients affected by soft tissue sarcoma.

**Conclusions:**

A delay in sarcoma diagnosis but not in starting treatment has been observed during the first year of the COVID-19 pandemic. Nevertheless, no difference in stage at diagnosis or in terms of survival has been observed.

## Introduction

Since late 2019, a new coronavirus, the severe acute respiratory syndrome coronavirus 2 (SARS-CoV-2), has emerged, causing the coronavirus disease (COVID-19), characterized by a wide range of symptoms, from completely asymptomatic cases to life-threatening respiratory failure (1–3). The rapid spread of COVID-19 disease worldwide led to the declaration of a pandemic by the World Health Organization (WHO) on 11 March 2020, to the rapid reorganization of social and work activities in many countries, and to the declaration of a lockdown to contain the spread of the infection (4). Italy was the first of the Western countries to be severely affected, and on 9 March 2020, a national lockdown was declared (5).

From the healthcare point of view, there was a rapid reorganization, with only the strictly necessary activities maintained, in order both to focus on the care of COVID-19 patients and to reduce the risk of contracting the virus (6, 7). Several effects of the pandemic have been observed in the field of oncology. The first is the worldwide reduction in the number of screenings for more than 80% for breast and cervical cancer, from 28% to 100% for colorectal cancer, more than 70% for prostate cancer, and more than 50% for lung cancer compared to the average for the same period of time in the years before the pandemic (8–10). Consequently, fewer diagnoses have been reported in 2020, and the number of consultations for advanced cancer or complications due to the extension of the disease has increased (11, 12). In a study conducted by Ferrara and colleagues in Central and Northern Italy, a 44.9% reduction in new cancer diagnoses in 2020 was registered, with skin melanoma being the most affected tumor with a 56.7% reduction, followed by colorectal cancer (46.6%), prostate cancer (45%), and bladder cancer (43.6%) (13). Several studies have shown similar data, with a reduction in new cancer diagnoses in spring 2020 and a recovery to 2019 numbers in the second half of the year (14–21). In literature, different studies analyzed whether there was a diagnostic delay due to the COVID-19 pandemic in different cancer types, showing in most cases that a high standard of care had been maintained during the COVID-19 peak of the pandemic, with for example no delay between diagnosis and treatment in head and neck cancers and no diagnostic or treatment onset delay in lung cancers (12, 22). On the other hand, a report showed a treatment delay in breast cancer due to the pandemic outbreak (23). Similar data are currently not uniformly collected, probably due to the subjective judgement in considering the onset of a suspected symptom by the patient, as opposed to the objective data on the number of new cases and the stage at diagnosis. Based on the considerations above, an increase in cancer deaths is expected in the coming years due to the reduction in the number of screening exams, the reduction of diagnosis in 2020, and the diagnostic or the start of treatment delay potentially caused by the pandemic (24, 25).

Sarcomas are rare tumors that often affect a young population. Due to both the rarity of the disease and the frequent association with sports injuries regarding the localizations of the limbs, the time to diagnosis is commonly known to be longer than for other malignancies (26). No data are available in the literature on the impact of the COVID-19 pandemic on diagnostic delay in sarcoma patients. However, literature data showed a reduction in the number of surgical and second opinion accesses in a musculoskeletal oncology unit during the pandemic compared to the previous year (27). Moreover, delays in surgical management related to COVID-19 led to higher morbidity in orthopedic oncology patients (28).

In this study, we aimed to retrospectively analyze whether there was a diagnostic delay due to the COVID-19 pandemic in patients with soft tissue sarcomas (STS), bone sarcomas (BS), or aggressive benign musculoskeletal diseases (ABMD) during the first year of the pandemic compared to the year before. We also analyzed the total number of consultations and of new diagnoses, and the impact on survival.

## Materials and methods

This is a single-center retrospective study based on a consecutive clinical series of patients referred to the Multidisciplinary Sarcoma Outpatient Clinic of the Regina Elena National Cancer Institute in Rome during the first year of the COVID-19 pandemic and during the 12 months before. The clinical cases were extracted from the registry of patients followed at our institute and coded within the international European Reference Network on Rare Adult Cancers (EURACAN) database whose clinical data were collected according to standardized and coded criteria (29).

Inclusion criteria were having performed the first outpatient visit in our institution between 9 March 2019 and 8 March 2021 and the histological diagnoses obtained in the Pathological Anatomy Laboratory of our institute of STS, BS, or ABMD (giant cell tumors of bone, aggressive fibromatosis, and pigmented villonodular tenosynovitis). The histological diagnosis had to be confirmed after centralized revision at our institution for patients whose initial diagnosis had been made in other centers. Exclusion criteria were all histological malignancy different from the mentioned above and the lack of clinical data.

The clinical data collected from the medical records were demographic data (age at diagnosis, sex, educational grade, professional occupation, civil status, and childhood); the date of the first symptom for which the patient judged it necessary to refer to a healthcare professional, of the start of the diagnostic workup, of the first instrumental examination, of the first oncological outpatient consultation at our institution, of the first histological diagnosis and the definitive diagnosis for patients for whom centralized histological revision was required, of the therapeutic decision, and of the start of the first treatment; where the diagnosis has been done; whether the diagnostic workup has been done in public or private clinics; the type of diagnosis; the extent of disease; the type of treatment; the overall survival (OS) and progression-free survival (PFS).

The primary objective was to determine whether the COVID-19 pandemic resulted in a diagnostic delay (time from first symptom to definitive histological diagnosis) compared to a control group diagnosed during the 12 months before the pandemic. The secondary objectives were to determine whether, during the first year of the COVID-19 pandemic compared to the previous 12 months, a greater number of more advanced diagnoses, a variation in the number of diagnoses, a variation in the number and type of first oncological consultation, a delay in patient care (i.e., time from clinical onset reported by the patient to the start of diagnostic workup, to the first diagnostic examination, to the first consultation at our institution, to the histological diagnosis, to the therapeutic decision, and to the start of treatment), and an impact on outcomes (PFS and OS) were observed.

Patients were classified into the Control group, if the first histological diagnosis had been made between 9 March 2019 and 8 March 2020, or the Covid group, if the first histological diagnosis had been made between 9 March 2020 (starting date of the national lockdown in Italy) and 8 March 2021. Only patients for whom the date of the first symptom and of the definitive diagnosis was available were analyzed for the diagnostic and therapeutic delay, the total number of new diagnoses, the stage of disease, and the survival analysis. All patients who accessed for a first outpatient visit were included in the analysis of the number and type of consultations.

The study was conducted according to the Declaration of Helsinki and was approved by the local Ethics Committee under the number 1676/22 and the acronym SarCorD Study (SARcoma CORonavirus diagnostic Delay).

### Statistical analysis

We reported the categorical variables through absolute and relative frequencies, whereas the continuous variables were reported through mean and standard deviations (SDs). We calculated the Kolmogorov–Smirnov normality test for all the continuous variables. The study groups were compared using Pearson’s Chi-square test or Student’s *t*-test, as appropriate. The Kaplan–Meier product-limit method and the Log-Rank test were used for estimating and comparing survival curves. Hazard ratios and their relative 95% confidence intervals were estimated for each variable using the univariate Cox proportional hazard model. A multivariate Cox model was than conducted considering the variables significant at univariate analysis. A *p*-value < 0.05 was considered statistically significant. Statistical analyses were carried out using SPSS version 21.0 (SPSS Inc., Chicago, Illinois, USA).

## Results

### Patients’ characteristics

**Figure 1:**
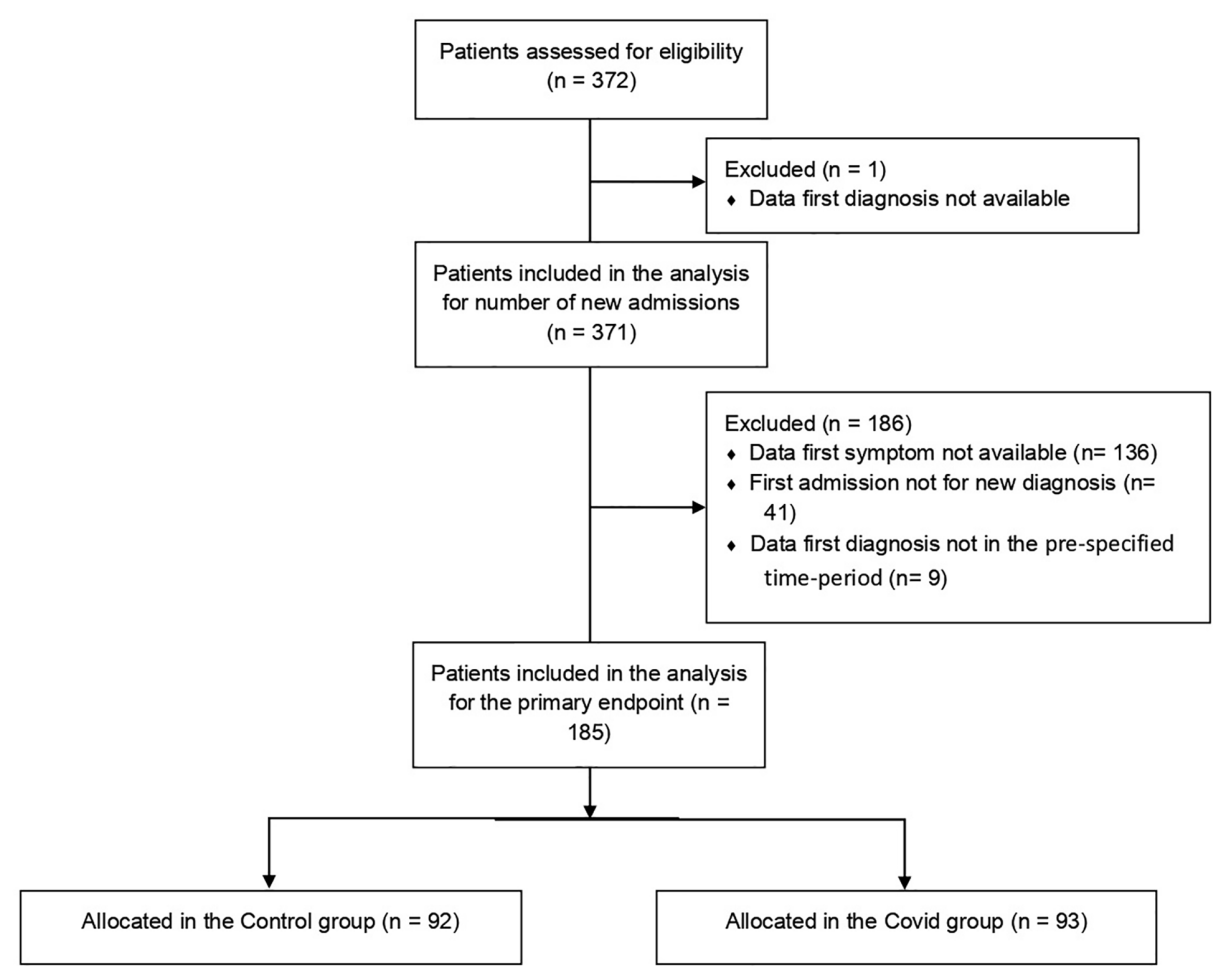
Consort diagram of patients included in the study. A total of 372 patients were selected from the EURACAN database, of whom 371 were eligible for secondary endpoint analysis of the number of new admissions. After exclusion of 186 patients not eligible for primary endpoint analysis, we included in the final analysis 185 patients, of whom 92 were in the Control group and 93 were in the Covid group.

A total number of 372 patients were referred to the Multidisciplinary Sarcoma Outpatient Clinic of the Regina Elena National Cancer Institute in Rome from 9 March 2019 to 8 March 2021, 137 with the first histological diagnosis obtained during the first year of the COVID-19 pandemic (Covid group) and 234 during the 12 months before (Control group). One patient was excluded due to the lack of information about the date of histological diagnosis. For the primary endpoint analysis, we excluded patients for which we had no information about the date of the first symptom in the clinical records, patients whose first admission to our institution was for a reason other than a new diagnosis, and patients with the first histological diagnosis obtained not in the pre-specified time period (**Figure 1**). Overall, 185 patients were eligible for the final analysis, 93 in the Covid group and 92 in the Control group. The mean age at diagnosis was 56.1 [standard deviation (SD) 17.6] and 53.9 (SD 18.0) in the Covid and Control group, respectively. The most frequent diagnosis was STS, accounting for 66.7% and 63.0% in the Covid and Control group, respectively, followed by BS in 23.7% and 20.7%, respectively, and other histology in 9.8% and 16.4% of the cases, respectively. Socio-demographic and clinical patients’ characteristics were comparable in the two study groups as reported in **Table 1**.

**Table 1:** Patients’ characteristics in Control and Covid groups.

N = 185		Control group	Covid group	χ^2^
		N = 92	N = 93	p-value
		N (%)	N (%)	
Age	(mean ± SD)	53.9 ± 18.0	56.1 ± 17.6	0.389*
Gender	M F	60 (65.2) 32 (34.8)	53 (57.0) 40 (43.0)	0.251
Educational level	Until first level of secondary school Equal or upper than second level of secondary school	31 (37.8) 51 (62.2)	26 (31.0) 58 (69.0)	0.353
Occupational status	No Yes	45 (54.9) 37 (45.1)	43 (51.2) 41 (48.8)	0.634
Civil status	Single/divorced/widowed Married/cohabiting	33 (41.3) 47 (58.8)	34 (43.0) 45 (57.0)	0.819
Sons	No Yes	17 (30.9) 38 (69.1)	20 (32.3) 42 (67.7)	0.876
Type of facility where diagnosis was made	Public Privat or private + public	39 (72.2) 15 (27.8)	52 (74.3) 18 (25.7)	0.797
Institution where diagnosis was made	Elsewhere Our institution	13 (14.9) 74 (85.1)	22 (24.4) 68 (75.6)	0.113
Type of first treatment	Chemotherapy alone Radiotherapy alone Surgery Chemoradiotherapy Electrochemotherapy Best supportive care Follow-up	27 (30.7) 5 (5.7) 46 (52.3) 2 (2.2) 1 (1.1) 0 (0.0) 7 (8.0)	32 (34.8) 0 (0.0) 47 (51.1) 2 (2.2) 0 (0.0) 2 (2.2) 9 (9.8)	0.168
Extension of the disease	Localized Metastatic	76 (83.5) 15 (16.5)	81 (88.0) 11 (12.0)	0.380
Hystotype	Soft tissue sarcoma Bone sarcoma Benign muscoloscheletal aggressive disease Gastrointestinal stromal tumor Kaposi sarcoma	58 (63.0) 19 (20.7) 9 (9.8) 2 (3.3) 3 (3.3)	62 (66.7) 22 (23.7) 6 (6.5) 2 (2.2) 1 (1.1)	0.709

*Student’s t-test.

### Number and type of new admissions

Out of the 372 patients enrolled, 371 were evaluable for the total number and type of visit. The total number of admissions was lower in the Covid group compared to the Control group (N = 137 *vs*. N = 234). The major impact of the decreased number of visits was observed during the first trimester (March–May 2020 *vs*. March–May 2019) of the pandemic (N = 21 *vs*. N = 81 in the Covid and Control groups, respectively) and during the fourth trimester (December–February 2020 *vs*. December–February 2019, N = 23 *vs*. N = 52), while we observed a recovery in the number of visits to an amount comparable to that of the Control group during the trimester June–August (N = 36 *vs*. N = 46) and September–November (N = 56 *vs*. N = 55). The variation in the number of admissions was statistically significant with a *p* < 0.001 (**Supplementary Table 1**).

We observed a variation in the number of the different types of visits, with 108 *vs*. 147 consultations for new diagnosis in the Covid and Control groups, respectively, 0 *vs*. 5 second opinion, 12 *vs*. 48 access for patients already followed in other centers who were admitted to our institution to continue treatment/follow-up, and 17 *vs*. 34 patients who performed single access for biopsy or histological examination (p = 0.003) (**Supplementary Table 2**).

Considering the group of 185 patients evaluable for the primary endpoint, we observed fewer new diagnoses in the first trimester of the pandemic from March to May (14 *vs*. 33 in the Covid and in the Control groups, respectively). In the trimester June–August and September–November, the number of new diagnoses in the Covid group exceeds that of the same trimester during the year before the pandemic (33 *vs*. 22 and 32 *vs*. 20, respectively). In the trimester from December to February, the number of new diagnoses was similar (14 *vs*. 17). The differences reported were statistically significant with a p = 0.005 (**Supplementary Table 3**).

### Diagnostic and therapeutic delay

**Figure 2:**
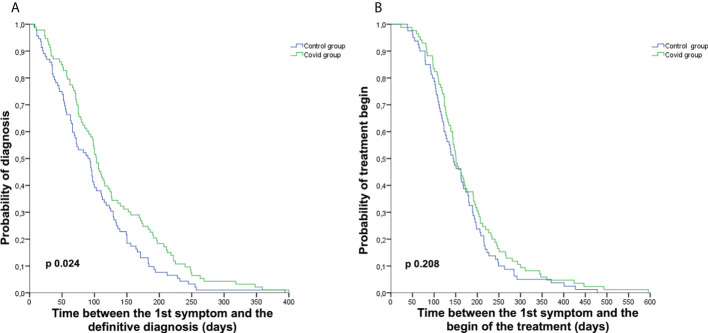
Kaplan–Meier curves representing diagnostic and treatment delay. Kaplan–Meier curves representing the time from the first symptom to definitive diagnosis **(A)** and from the first symptom to the start of the first treatment **(B)** in the Covid group (green) and the Control group (blue).

Considering the cohort of 185 patients evaluable for the primary endpoint, we observed a diagnostic delay in the Covid group with a median time from the first symptom to the definitive histological diagnosis of 103.00 days [95% confidence interval (CI) 92.77–113.23] *vs*. 90.00 days (95% CI 69.49–110.51) in the Control group (*p* = 0.024). Nevertheless, we did not observe a delay between the appearance of the first symptom to the first treatment beginning 151.00 days (95% CI 132.94–169.06) *vs*. 144.00 days (95% CI 120.33–167.67) in the Covid and Control groups, respectively (*p* = 0.208) (**Figure 2**). The difference in time between the first symptom and the first histological diagnosis (different from definitive diagnosis for patients requiring centralized histological revision) was also statistically significant, with a median time of 99.00 days (95% CI 78.38–119.62) *vs*. 72.00 days (95% CI 52.35–91.65) in the Covid and Control groups, respectively (*p* = 0.022). The evaluations of time from the first symptom to the beginning of diagnostic workup, to the first instrumental examination, to first access at our institution, to therapeutic decision, and from definitive histological diagnosis to therapeutic decision and treatment start did not show a delay in the Covid group compared to the Control group.

The multivariate analysis showed that diagnosis during the Covid period (HR 0.72, 95% CI 0.53–0.97, *p* = 0.034) and occupational status (HR 0.63, 95% CI 0.46–0.88, *p* = 0.006), with a longer time to diagnosis for professionally employed patients, are predictive of diagnostic delay (**Table 2**).

**Table 2:** Univariate and multivariate analysis for the diagnostic delay.

		Univariate Cox regression model		Multivariate Cox regression model	
		HR (95% CI)	p-value	HR (95% CI)	p-value
Group of diagnosis	Covid vs. Control	0.72 (0.53–0.96)	0.025	0.72 (0.53–0.97)	0.034
Age		1.00 (0.99–1.01)	0.957		
Gender	F vs. M	0.91 (0.68–1.23)	0.555		
Educational level	Equal to or higher than second level of secondary school vs. Until first level of secondary school	1.04 (0.75–1.44)	0.802		
Occupational status	Yes vs. No	0.63 (0.45–0.87)	0.005	0.63 (0.46–0.88)	0.006
Civil status	Yes vs. No	0.86 (0.63–1.19)	0.372		
Sons	Yes vs. No	0.67 (0.45–1.00)	0.052		
Type of facility where diagnosis was made	Private or private + public vs. Public	1.20 (0.80–1.79)	0.385		
Institution where diagnosis was made	Our institution vs. elsewhere	1.25 (0.85–1.84)	0.251		

HR, hazard ratio; CI, confidence interval.

Although the diagnostic delay was observed in all the trimesters, only during the trimester from September to November it was statistically significant, with a median time to definitive diagnosis of 99.00 days (95% CI 88.45–109.55) *vs*. 70.00 days (95% CI 59.53–80.47) in the Covid and Control groups, respectively (*p* = 0.035, **Table S4** and **Supplementary Figure 1**).

### Survival analysis

In the cohort of 185 patients, we did not observe a difference in stage at diagnosis in the Covid and in the Control groups, with 12.0% *vs*. 16.5% of patients metastatic at diagnosis in the two groups, respectively (*p* = 0.380, **Table 1**).

**Figure 3:**
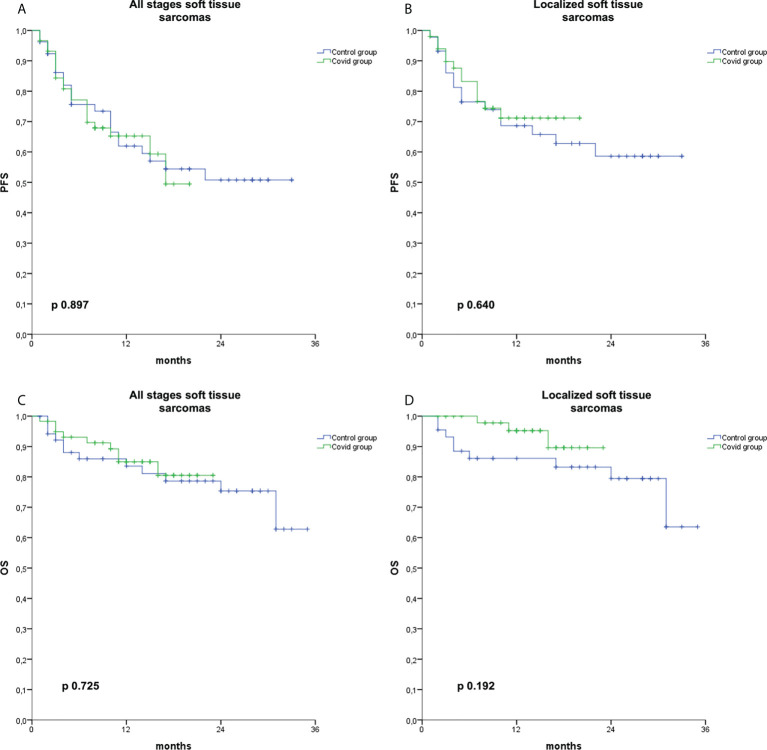
Kaplan–Meier curves for PFS and OS in soft tissue sarcomas. Kaplan–Meier curve for PFS in soft tissue sarcomas irrespective of the stage at diagnosis **(A)**, for PFS in localized soft tissue sarcomas **(B)**, for OS in soft tissue sarcomas irrespective of the stage at diagnosis **(C)**, and for OS in localized soft tissue sarcomas **(D)** in the Covid group (green) and the Control group (blue).

We also analyzed survival in the group of patients affected by STS, but not in other histologies, due to the small sample size. With a data cutoff on 28 February 2022, after a median follow-up of 13 months (range 1–24) and 23 months (range 1–36) in the Covid and Control groups, respectively, we did not observe any difference in terms of PFS or in terms of OS (**Figure 3**) in the subgroup of patients affected by STS irrespective of the stage at diagnosis (PFS: *p* = 0.897; OS: *p* = 0.725) or in limited stage STS (PFS: *p* = 0.640; OS: *p* = 0.192).

## Discussion

The emergence of the COVID-19 pandemic caused sudden changes in social organization and healthcare facilities. At the beginning, the healthcare system was not ready to cope with such a situation without jeopardizing its remaining activities. For this reason, hospitals have been reorganized, prioritizing only activities considered not deferrable and the treatment of COVID-19 patients. Most oncology activities were classified as non-deferrable; thus, anticancer treatment continued in most hospitals even during the pandemic. However, some changes were also observed in the oncology field, particularly with a larger use of schedules with longer intervals, a preference for the use of oral treatments over intravenous ones, a larger use of neoadjuvant therapy in order to delay surgery, the use of hypofractionated radiotherapy, and an earlier shift towards supportive care (7, 30). Most of these measures were taken based on expert opinions and not on objective data (31). However, it must be considered that the extent of the measures taken is commensurate with the local situation, the type of hospital, and the available resources, with a greater impact expected especially on hospitals with Covid wards (31). In addition, the psychological impact of the pandemic outbreak on the general population must be considered (32, 33). In particular, the fear of contracting COVID-19 has led patients in many medical fields to postpone investigations even in the presence of new symptoms. This, in addition to the reorganization of hospital activities, led us to observe a reduction in the number of cancer screenings, a reduction in the number of diagnoses during the first year of the pandemic, and, in some cases, a delay in taking care of patients (8, 9, 11–21, 23).

Few data are available in the literature regarding the impact of the COVID-19 pandemic on patients with STS, BS, and ABMD. As previously reported, in our orthopedic oncology unit during the first pandemic peak, priority was established for outpatient access and surgery, prioritizing new diagnoses of intermediate/high-grade sarcoma, orthopedic complications of bone metastases or rare benign aggressive tumors, recurrences during follow-up, and postoperative complications (34). This attitude has contributed to maintaining a high standard of care and avoiding delays in patient care.

To our knowledge, this study is the first conducted in this patient setting to investigate a possible diagnostic delay related to the pandemic outbreak. In our study, we observed a diagnostic delay during the first year of the pandemic in patients with STS, BS, or ABMD, with a time to diagnosis approximately 13 days longer than in the pre-pandemic period. As observed, the time to diagnosis for this type of disease was rather long even before the pandemic, due to the type of disease that often affects a young population and rarely affects vital organs, (26). Moreover, the first symptom is often associated with a sports injury with consequent underestimation of the symptoms (26). The moment that determined the diagnostic delay has not been identified, as no statistically significant delays were observed at the other times of the diagnostic workflow. However, in a multivariate analysis, we identified, in addition to the diagnosis during the pandemic, the occupational aspect as an independent prognostic factor for the diagnostic delay, with a longer time to diagnosis for professionally employed patients. The greatest impact of the diagnostic delay was not during the first peak of the pandemic (March–May 2020), but in the September–November trimester. To explain this, we must consider that the Covid and Control groups were established based on the date of the first histological diagnosis. Therefore, patients whose diagnosis was made in the first trimester of the pandemic had started the diagnostic process in the pre-pandemic period, whereas the delay is more evident among patients who had their first symptom during the pandemic. Interestingly, although a diagnostic delay was observed, there was no delay in starting treatment. This finding suggests that after the patient was correctly referred to a recognized experienced center with specific multidisciplinary skills, the diagnostic and therapeutic process took place without any delays compared to the year before the pandemic. The type of facility where the study was conducted must also be considered in this context. In fact, we are a National Cancer Institute where no unit has ever been converted into a Covid ward. Moreover, the first pandemic peak affected Northern Italy devastatingly, but was much more limited in the remaining areas of the country.

As also observed in other studies investigating the number of new diagnoses of solid tumors, in our study, we observed a decrease in new diagnoses during the first trimester of the pandemic, with a recovery in the following months (12, 14–21). We also analyzed the type of the first visit made and we noticed a reduction in the number of all types of accesses. In fact, during the first year of the pandemic, we observed a reduction in the number of patients already followed in other centers who came to our institutions to continue treatment and a reduction to zero in the number of second opinion. These data were probably determined by patients’ need to reduce hospital admissions to reduce the risk of infection, as our center did not set a limit on the type of first admission. This finding is a preoccupying aspect as regards the quality of care in patients affected by rare cancer, if we consider that in Central-Southern Italy only four institutes are EURACAN-recognized referral centers for the treatment of rare tumors, including the Regina Elena National Cancer Institute in Rome (29).

Fortunately, no increase in advanced disease has been observed regarding the stage at diagnosis. Furthermore, survival curves were overlapping in the subgroup of patients affected by STS. This can be explained both by the fact that there was no delay in the start of treatment and by the fact that sarcomas are often slow diseases at an early stage. A longer follow-up will certainly be necessary, considering, at the current state, the short follow-up for the patients included in this analysis, especially for those in the Covid group.

The study presented has the limitation of being a retrospective study, resulting in the loss of some data, especially regarding the date of the first symptom, which is often not included in the routinely collected clinical history. Moreover, the date of the first symptom onset is difficult to evaluate mainly for two reasons. The first is that it is based not on objective data but on the patient’s memory. The second concerns the type of disease, as the lesion often appeared a long time before but was underestimated by the patient. To overcome this issue, we used the definition of first symptom for which the patient found it necessary to consult a doctor, but this definition has the disadvantage of being very subjective. The advantage of this study is that it is the first conducted on patients with STS, BS, and ABMD. This study was conducted in a systematic manner, as the number of accesses was extracted from the EURACAN database, with the consequent reduction of the risk of missing patients. As the study is monocentric, the sample size is limited. It would be interesting to conduct a similar multicentric study in order both to increase the sample size and to assess whether the impact of the pandemic differed according to geographical area or hospital type.

In conclusion, to our knowledge, this is the first and largest study to have analyzed the pandemic-related diagnostic delay in sarcomas and ABMD. A pandemic-related diagnostic delay was observed, but not a delay in treatment onset. Fortunately, the diagnostic delay resulted in neither an increase in late-stage diagnoses nor an impact on survival, although longer follow-up is necessary. A reduction in the number of accesses was observed during the first year of the pandemic, particularly in the first trimester from March to May 2020, both in terms of new diagnoses and for patients already followed elsewhere who came to our center to continue treatment or for a second opinion.

## Data availability statement

The raw data supporting the conclusions of this article will be made available by the authors upon reasonable request.

## Ethics statement

This study was reviewed and approved by Comitato Etico IFO-Regina Elena. Written informed consent from the participants was not required in accordance with the national legislation and the institutional requirements.

## Author contributions

CEO, SV, BR, MCC and VF conceived the study. CEO, BR, FS, MCC, AC and VF wrote the project. CEO, FN, GM, DM and EC were involved in data collection. FS performed statistical analysis. CEO, SV, BR, RB, GC and VF interpreted the results. CEO wrote the article. All the authors revised and approved the final version. All authors contributed to the article and approved the submitted version.

## Funding

This study was supported by funds from the COMETA project, funded by the Soka Gakkai Italia association.

## Conflict of interest

The authors declare that the research was conducted in the absence of any commercial or financial relationships that could be construed as a potential conflict of interest.

The reviewer DM declared a past co-authorship with the author SV and a shared parent affiliation with the author AC to the handling editor at the time of review. The author CEO declared past co-authorships with the Editor RG.

## Publisher’s note

All claims expressed in this article are solely those of the authors and do not necessarily represent those of their affiliated organizations, or those of the publisher, the editors and the reviewers. Any product that may be evaluated in this article, or claim that may be made by its manufacturer, is not guaranteed or endorsed by the publisher.

## Abbreviations

STS, soft tissue sarcoma; BS, bone sarcoma; ABMD, aggressive benign musculoskeletal disease; EURACAN, European Reference Network on Rare Adult Cancers.
