# Supplementary material for: The impact of the COVID-19 pandemic on diagnosis and treatment of patients with soft tissue and bone sarcomas or aggressive benign musculoskeletal diseases: A single-center retrospective study (SarCorD study)
Source: Front Oncol. 2022 Sep 20;12:1000056. doi: 10.3389/fonc.2022.1000056 (PMC9559379; doi:10.3389/fonc.2022.1000056)
Supplement: Supplementary file 1 [file DataSheet_1.pdf]

Supplementary Material

Supplementary Table 1. Number of first visits per trimester. 2
Supplementary Table 2. Number of new admissions according to the type of visit. 2
Supplementary Table 3. Number of new diagnoses per trimester. 2
Supplementary Table 4. Diagnostic delay in the different trimesters. 3
Supplementary Figure 1. Diagnostic delay in the different trimesters. 4

Supplementary Table 1. Number of first visits per trimester.

**Trimester definitive diagnosis**	**Number of first visits per trimester Control group** N (%)	**Number of first visits per trimester Covid group** N (%)	**p value**
March-May	81 (34.6)	21 (15.3)	**< 0.001**
June-August	46 (19.7)	36 (26.7%)	
September-November	54 (23.1)	55 (40.1)	
December-February	52 (22.2)	23 (16.8)	

Missing data for 1 patient in Control group and for 2 patients in Covid group.

Supplementary Table 2. Number of new admissions according to the type of visit.

**Type of consultation**	**Number of visits Control group** N (%)	**Number of visits Covid group** N (%)	**p value**
New diagnosis	147 (62.8)	108 (78.8)	**0.003**
Second opinion	5 (2.1)	0 (0.0)	
Center change	48 (20.5)	12 (8.8)	
Single access	34 (14.5)	17 (12.4)	

Supplementary Table 3. Number of new diagnoses per trimester.

**Trimester definitive diagnosis**	**Number of new diagnoses per trimester Control group** N (%)	**Number of new diagnoses per trimester Covid group** N (%)	**p value**
March-May	33 (35.9)	14 (15.1)	**0.005**
June-August	22 (23.9)	33 (35.5)	
September-November	20 (21.7)	32 (34.4)	
December-February	17 (18.5)	14 (15.1)	

Supplementary Table 4. Diagnostic delay in the different trimesters.

**Trimester definitive diagnosis**	**Time to diagnosis in Control group** Median time in days (95% CI)	**Time to diagnosis in Covid group** Median time in days (95% CI)	**p value**
March-May	66.00 (23.04-108.96)	98.00 (59.61-136.39)	0.235
June-August	115.00 (76.12-153.88)	121.00 (93.28-148.72)	0.659
September-November	70.00 (59.53-80.47)	99.00 (88.45-109.55)	**0.035**
December-February	72.00 (15.87-128.13)	91.00 (61.64-120.36)	0.843

Abbreviation: CI = Confidence interval.

Supplementary Figure 1. Diagnostic delay in the different trimesters.

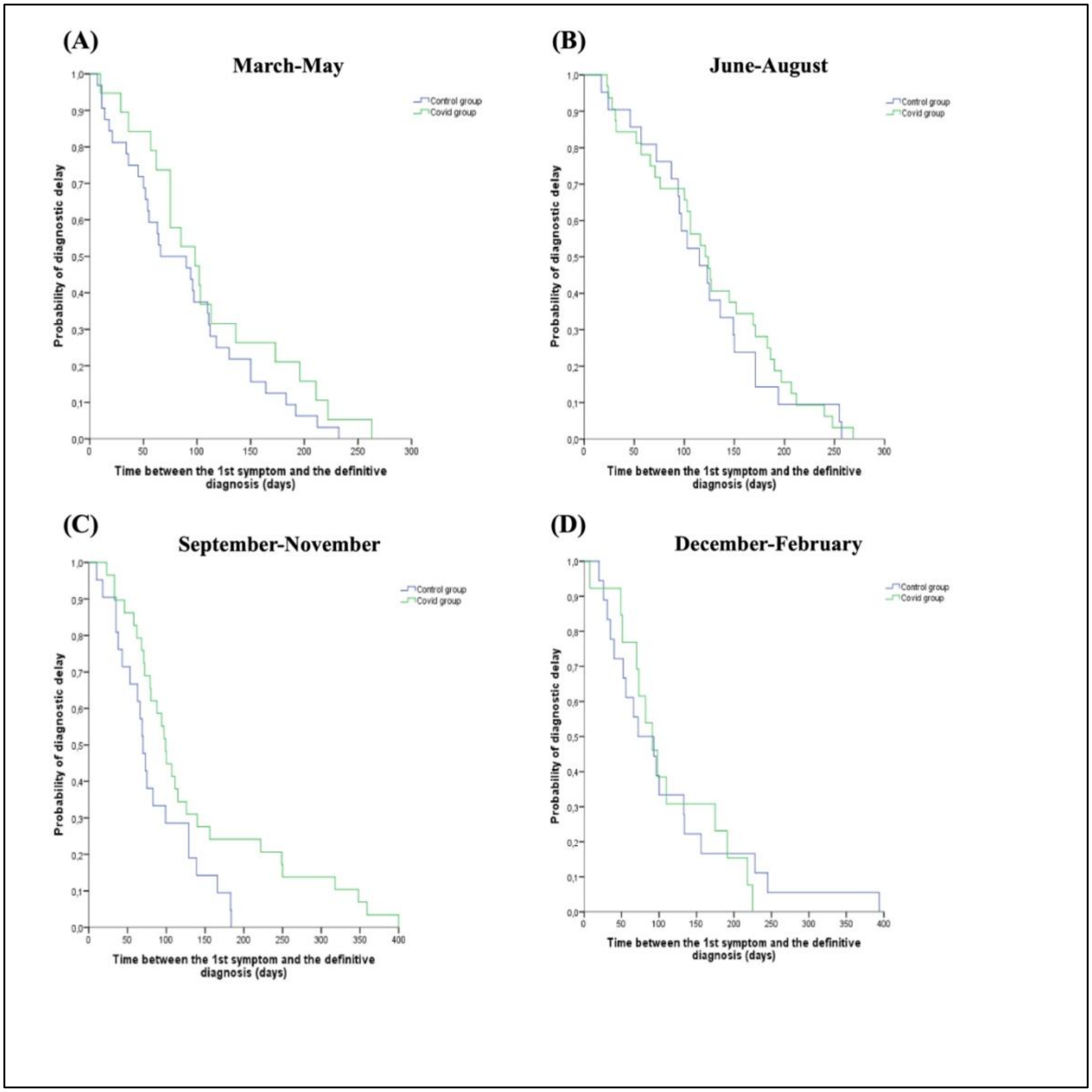

Kaplan-Meier curves representing the time from first symptom to definitive diagnosis in the trimester March-May (A), June-August (B), September-November (C), and December-February (D) in Covid group (green) and Control group (blue).
